# Perceptions of the safety, development, and approval process of severe acute respiratory syndrome coronavirus 2 (SARS-CoV-2) vaccines among individuals with Affordable Care Act health insurance in Central Texas at the time of initial vaccine availability

**DOI:** 10.3389/fpubh.2025.1446984

**Published:** 2025-06-24

**Authors:** Carlos Lopez Bray, Richard Taylor, Naomi Tamez, Wesley Durkalski, John R. Litaker

**Affiliations:** ^1^Office of Population Health, Sendero Health Plans, Inc., Austin, TX, United States; ^2^Undergraduate Public Health Program, The University of Texas at Austin, Austin, TX, United States; ^3^Former Chief Executive Officer, Sendero Health Plans, Inc., Austin, TX, United States; ^4^Office of Population Health and Science, The Litaker Group, LLC, Austin, TX, United States

**Keywords:** COVID-19, vaccines, Affordable Care Act, vaccine attitudes, vaccine beliefs, Sendero Health Plans

## Abstract

Sendero Health Plans, an Affordable Care Act (ACA) health insurance company, conducted a cross-sectional survey in December 2020 to assess individual perceptions of the COVID-19 vaccine development and approval processes and their plan to obtain the COVID-19 vaccine in Central Texas at the time of initial availability. A logistic regression model was developed to identify factors associated with individual plans to obtain a vaccine when it became available. A total of 500 (77.25%) of the 645 respondents in this analysis planned to obtain a COVID-19 vaccine when it became available. The logistic regression model was statistically significant [χ^2^(19) = 314.41, *p* < 0.001]. Plans to obtain a COVID-19 vaccine were significantly associated with perceptions of vaccine safety (POR: 23.45, *p* = 0.001, 95% CI: 3.10, 58.34), vaccine protectiveness (POR: 15.55, *p* < 0.001, 95% CI: 3.55, 68.06), and transparency of the authorization process (POR: 1.33, *p* = 0.024, 95% CI: 1.15, 7.15). Perceptions regarding the safety, protectiveness, and authorization process of the COVID-19 vaccines are associated with individual plans to obtain the vaccines. This study provides insights into factors that influence vaccination intent and key barriers affecting vaccine hesitancy during a public health crisis.

## 1 Introduction

The vaccine research, development, and approval process is robust. On average, vaccine development can take up to a decade to complete. The clinical trial process includes investigation of the compound in human subjects, safety and efficacy of the vaccine, and post-marketing data once the vaccine is available to the general population. This process requires monitoring, oversight, and strict compliance with safety protocols and regulations before approval is granted (1). In 2020, the US government initiated Operation Warp Speed to hasten the development and clinical trials process for the severe acute respiratory syndrome coronavirus 2 (SARS-CoV-2) vaccine. Novel messenger ribonucleic acid (mRNA) technology was used to develop two vaccines, the BNT162b2 vaccine manufactured by Pfizer-BioNTech and the mRNA-1273 vaccine manufactured by Moderna. As part of Operation Warp Speed, Moderna received $483 million from the US government to support initial Phase 1 clinical trials and an additional $472 million a few months later to support late-stage clinical development (2). Pfizer-BioNTech did not receive funding from Operation Warp Speed; however, they received $445 million from the government of the Federal Republic of Germany (3).

On December 11, 2020, and December 18, 2020, the US Food and Drug Administration (FDA) granted emergency use authorization (EUA) for both the Pfizer and Moderna mRNA coronavirus disease 2019 (COVID-19) vaccines, respectively (4). This was the first time that the FDA had granted EUA for human vaccines and the first time for mRNA technology. Enabling legislation allows the US Secretary of Health and Human Services to certify EUA if it is reasonable to believe, based on the totality of scientific evidence available, including data from adequate and well controlled clinical trials, that EUA is in the best interests of the public (5, 6). Specific considerations for EUA certification include:

1. The pathogen being protected against is capable of causing *serious or life-threatening* harm;
2. The EUA product *may be effective* in diagnosing, treating, or preventing the specific disease, serious, or life-threatening condition in which an emergency has been declared;
3. The known benefits of the EUA product outweigh the known risks or potential risks of consuming the product; and.
4. There are no *adequate, approved, or available alternatives* to the EUA product to treat the condition (6).

The prescribed EUA process provides an expedited authority to protect lives from serious or life-threatening disease during extraordinary circumstances for which an approved product is not currently available but in which a product demonstrates scientific safety and efficacy is available. The two mRNA COVID-19 vaccines were deemed to have met these safety and efficacy criteria and were authorized by the US Secretary for Health and Human Services.

Despite the approval and distribution of the Pfizer-BioNTech and Moderna mRNA vaccines and despite efficacy and effectiveness showing reduced burden of COVID-19 disease, vaccine hesitancy ensued (7, 8). In the case of COVID-19, as with other vaccines, vaccine hesitancy has its roots in sociodemographic characteristics, perceived risk of disease, and vaccine safety (9, 10). However, COVID-19 vaccines faced additional challenges due to a lack of knowledge by the general public about mRNA technology, no previous use of mRNA vaccine technology in an approved human vaccine, a growing public misunderstanding and mistrust of mRNA technology, and concern about the expedited development and approval process for the mRNA vaccines despite the strong safety profile of the two mRNA vaccines (11).

The purpose of this study was to identify associations between an individual’s current plans to obtain the COVID-19 vaccine at the time of initial availability and their perceptions of the COVID-19 development and approval processes, including: (1) mRNA vaccine safety; (2) mRNA vaccine protection; (3) transparency associated with the federal EUA process; and (4) the expedited timeframe to authorize vaccine under EUA.

## 2 Materials and methods

Sendero Health Plans, Inc. (Sendero) electronically administered a cross-sectional survey to eligible Sendero members from December 24, 2020, through December 31, 2020 using the online Qualtrics platform (Qualtrics, Provo, UT, United States). The survey administration period coincided with the first week of COVID-19 vaccine availability to the general public in Austin, Texas. Eligible survey participants were adult (18 years or older) head-of-household members enrolled in a Patient Protection and Affordable Care Act (ACA) plan. Head-of-household members were the primary policyholder. Survey participation was voluntary, and respondents were offered a $25 gift card upon submission of the completed survey. The survey and all subsequent communications were administered in both English and Spanish.

The survey requested information related to members’ plans to obtain the COVID-19 vaccine and their perceptions of the research, development, approval processes, and timing of COVID-19 vaccine delivery. The primary outcome of interest was the respondents’ response to the statement, “I plan to get the COVID-19 vaccine when it is available.” Response options (“yes,” “no,” “prefer not to answer,” and “unsure”) were subsequently categorized as a dichotomous response (“yes,” or “not yes”) for analysis. To assess respondents’ beliefs about the research and development process to create safe and protective COVID-19 vaccines, the transparency related to the FDA emergency use authorization process, and the time to authorize the COVID-19 vaccines, respondents were asked to provide their level of agreement with each of the following statements:

1. The research and development process has produced COVID-19 vaccines that are safe;
2. The research and development process has produced COVID-19 vaccines that will protect me;
3. The FDA emergency use authorization process for approving COVID-19 vaccines is transparent; and.
4. The time it took to authorize COVID-19 vaccines was appropriate.

The original five levels of agreement (strongly disagree, disagree, neutral, agree, or strongly agree) for each of these statements were subsequently stratified to three levels (disagree, neutral, or agree) for analysis to conserve statistical power.

Reported sociodemographic characteristics (e.g., age, sex at birth, race, ethnicity, highest level of education, annual household income) were obtained from a previously administered survey that included respondents of this survey. Detailed methods and results for this survey are described elsewhere (12). Race was collapsed into distinctly “White” and “Non-white” categories for any race representing less than 10% of the respondent sample. Observations containing “Prefer not to answer” or “Other” as responses to the question regarding annual income were removed from the analysis due to a quantifiable income that could be categorized. Descriptive statistics for categorical variables include level-specific frequencies, totals and percentages, and continuous variables include the mean, range, and standard deviation. Unadjusted logistic regression models were used to assess independent associations between selected independent variables and the outcome variable of interest. An adjusted logistic regression model, including only variables significantly associated with the outcome variable in unadjusted regression, was developed to identify the variables that most influence the outcome of interest while controlling for predictor variables. The threshold to determine statistical significance for adjusted and unadjusted logistic regression models was alpha (*α*) = 0.05. Unadjusted and adjusted prevalence odds ratios (POR) and corresponding 95% confidence intervals (95% CI), and *p*-values to assess the associations between each independent predictor variable and the dependent variable, and the chi-square statistic for the adjusted model and corresponding *p*-value are reported. Analyses were conducted using STATA (16.1, StataCorp LLC, College Station, TX).

## 3 Results

### 3.1 Demographic profile

A total of 737 (88.2%) of the 836 eligible members completed the survey. Of these, 645 (87.5%) remained in the analysis after data cleaning. Among the respondents, 500 (77.5%) planned to obtain the COVID-19 vaccine when it became available, and 145 (22.5%) did not plan to obtain the vaccine, were unsure, or preferred not to answer. Respondent mean age was 48.1 years (range: 21.4–86.3 years, SD ± 11.9). Most respondents were women (53.5%), of White race (85.6%), reported an annual household income between US $10,000–$29,999 (32.2%), and had a bachelor’s or graduate degree as their highest level of education (60.5%). There were 118 (18.3%) participants of Hispanic, Latino, or Spanish ethnicity. Demographic data are summarized in Table 1.

**Table 1:** Reported demographic and summary characteristics of survey respondents.

Characteristics of the Respondent Population	Respondents, *N* = 645
Sex	
Male	300 (46.5)
Female	345 (53.5)
Age in Years (Mean: 48.1 years, Range: 21.4–86.3, SD ± 11.9)	
18–34 years old	115 (17.8)
35–44 years old	157 (24.3)
45–54 years old	139 (21.6)
≥ 55 years old	234 (36.3)
Race	
White	552 (85.6)
Non-White	93 (14.4)
Ethnicity	
No, not of Hispanic, Latino, or Spanish origin	527 (81.7)
Yes, Hispanic, Latino, or Spanish origin	118 (18.3)
Education	
≤ High School	76 (11.8)
Some College, Technical School or Associate degree	179 (27.8)
Bachelor or Graduate Degree	390 (60.5)
Annual Household Income	
<$10,000	64 (9.9)
$10,000–$29,999	208 (32.2)
$30,000–$39,999	95 (14.7)
$40,000–$49,999	64 (9.9)
$50,000–$75,999	107 (16.6)
$76,000–$99,999	44 (6.8)
$100,000 or more	63 (9.8)
Plan to Obtain the COVID-19 Vaccine	
Yes	500 (77.52)
Not yes	145 (22.48)
The research and development process has produced COVID-19 vaccines that are safe	
Disagree	30 (4.7)
Neutral	198 (30.7)
Agree	417 (64.7)
The research and development process has produced COVID-19 vaccines that will protect me	
Disagree	30 (4.7)
Neutral	171 (26.5)
Agree	444 (68.8)
The FDA emergency use authorization process for approving COVID-19 vaccines is transparent	
Disagree	87 (13.5)
Neutral	294 (45.6)
Agree	264 (40.9)
The time it took to authorize COVID-19 vaccines was appropriate	
Disagree	94 (14.6)
Neutral	216 (33.5)
Agree	335 (51.9)

### 3.2 Perceptions about the COVID-19 vaccine safety, protectiveness, and authorization process

There were 417 (64.7%) respondents who agreed that the research and development process produced safe COVID-19 vaccines, 198 (30.7%) were neutral, and 30 (4.7%) disagreed. Among those who agreed or were neutral, 400 (95.9%) and 93 (46.9%) respondents planned to obtain the COVID-19 vaccine, respectively. Regarding the statement, “the research and development process has produced COVID-19 vaccines that will protect me,” 444 (68.8%) respondents agreed, 171 (26.5%) were neutral, and 30 (4.7%) disagreed. Among respondents who agreed or were neutral, 419 (94.4%) and 77 (45%) planned to obtain the COVID-19 vaccine when available, respectively. Most respondents who disagreed with statements regarding COVID-19 vaccine safety and protectiveness did not indicate that they planned to receive the vaccine once it became available.

When asked whether the FDA EUA process for approving COVID-19 vaccines was transparent, 264 (40.9%) respondents agreed with the statement, 294 (45.6%) were neutral, and 87 (13.5%) disagreed. Among those who agreed or were neutral, 244 (92.4%) and 214 (72.8%) planned to obtain the COVID-19 vaccine when available, respectively. Lastly, 335 (51.9%) respondents agreed with the statement, “the time it took to authorize COVID-19 vaccines was appropriate,” 216 (33.5%) were neutral, and 94 (14.6%) disagreed. Among those who agreed or were neutral, 311 (92.8%) and 147 (68.1%) planned to obtain the COVID-19 vaccine when available, respectively. Findings are summarized in Table 1.

### 3.3 Bivariate analysis

Results of unadjusted analyses are shown in Table 2. Being male, of Hispanic ethnicity, of White race, having a bachelor’s or graduate degree, and having an annual income of $10,000 or more were statistically significant independent predictors of plans to get a COVID-19 vaccine. Additionally, perceptions regarding the safety, protectiveness, transparency, and timeliness of the authorization process of the COVID-19 vaccines were significantly associated with the outcome variable.

**Table 2:** Results of adjusted and unadjusted logistic regression models of plans to receive the COVID-19 vaccine for possible predictor variables.

Variable	Total; *N* = 645	Yes; *n* = 500	Not Yes; *n* = 145	Yes vs. Not Yes			
				Unadjusted OR (95% CI)	*p*-value	Adjusted OR (95% CI)	*p*-value
Sex, n (%)							
Female	345 (53.5)	248 (49.6)	97 (66.9)	1.0			
Male	300 (46.5)	252 (50.4)	48 (33.1)	2.04 (1.39, 3.03)	< 0.001*	2.05 (1.16, 3.62)	0.014*
Age (years), n (%)							
18–34 years old	115 (17.8)	89 (17.8)	26 (17.9)	1.0			
35–44 years old	157 (24.3)	114 (22.8)	43 (29.7)	0.77 (0.44, 1.36)	0.371	–	–
45–54 years old	139 (21.6)	99 (19.8)	40 (27.6)	0.72 (0.41, 1.28)	0.265	–	–
≥ 55 years old	234 (36.3)	198 (39.6)	36 (24.8)	1.61 (0.91, 2.82)	0.099	–	–
Hispanic, Latino, or Spanish Ethnicity, n (%)							
Yes	118 (18.3)	81 (16.2)	37 (25.5)	1.0			
No	527 (81.7)	419 (83.8)	108 (74.5)	1.77 (1.14, 2.76)	0.011*	1.39 (0.71, 2.72)	0.340
White, n (%)							
No	93 (14.4)	51 (10.2)	42 (29)	1.0			
Yes	552 (85.6)	449 (89.8)	103 (71)	3.59 (2.26, 5.69)	< 0.001*	3.59 (1.74, 7.42)	0.001*
Education level, n (%)							
≤ High School	76 (11.8)	48 (9.6)	28 (19.3)	1.0			
Some College, Technical School or Associate degree	179 (27.8)	121 (24.2)	58 (40)	1.22 (0.69, 2.13)	0.493	1.11 (0.49, 2.48)	0.807
Bachelor or Graduate Degree	390 (60.5)	331 (66.2)	59 (40.7)	3.27 (1.90, 5.63)	< 0.001*	1.54 (0.71, 3.34)	0.275
Household income, n (%)							
<$10,000	64 (9.9)	39 (7.8)	25 (17.2)	1.0			
$10,000–$29,999	208 (32.2)	160 (32)	48 (33.1)	2.14 (1.18, 3.88)	0.013*	1.66 (0.70, 3.94)	0.253
$30,000–$39,999	95 (14.7)	74 (14.8)	21 (14.4)	2.26 (1.12, 4.54)	0.022*	1.86 (0.68, 5.03)	0.222
$40,000–$49,999	64 (9.9)	52 (10.4)	12 (8.3)	2.78 (1.24, 6.21)	0.013*	2.27 (0.73, 7.02)	0.156
$50,000–$75,999	107 (16.6)	82 (16.4)	25 (17.2)	2.10 (1.07, 4.12)	0.030*	0.88 (0.33, 2.36)	0.802
$76,000–$99,999	44 (6.8)	41 (8.2)	3 (2.1)	8.76 (2.45, 31.36)	0.001*	3.16 (0.53, 18.78)	0.206
$100,000 or more	63 (9.8)	52 (10.4)	11 (7.6)	3.03 (1.33, 6.89)	0.008*	0.95 (0.28, 3.21)	0.937
COVID-19 vaccines are safe, n (%)							
Disagree	30 (4.7)	7 (1.4)	23 (15.9)	1.0			
Neutral	198 (30.7)	93 (18.6)	105 (72.4)	2.91 (1.19, 7.09)	0.019*	1. 09 (0.32, 3.65)	0.892
Agree	417 (64.7)	400 (80)	17 (11.7)	77.31 (29.15, 205.05)	< 0.001*	8.71 (2.25, 33.66)	0.002*
COVID-19 vaccines are protective, n (%)							
Disagree	30 (4.7)	4 (0.8)	26 (17.9)	1.0			
Neutral	171 (26.5)	77 (15.4)	94 (64.8)	5.32 (1.78, 15.91)	0.003*	3.22 (0.82, 12.75)	0.095
Agree	444 (68.8)	419 (83.8)	25 (17.2)	108.94 (35.28, 336.35)	< 0.001*	13.45 (3.10, 58.34)	0.001*
COVID-19 vaccines authorization process was transparent, n (%)							
Disagree	87 (13.5)	42 (8.4)	45 (31)	1.0			
Neutral	294 (45.6)	214 (42.8)	80 (55.2)	2.87 (1.75, 4.69)	< 0.001*	2.36 (1.15, 4.86)	0.020*
Agree	264 (40.9)	244 (48.8)	20 (13.8)	13.07 (7.03, 24.30)	< 0.001*	2.87 (1.15, 7.15)	0.024*
COVID-19 vaccines authorization time was appropriate, n (%)							
Disagree	94 (14.6)	42 (8.4)	52 (35.9)	1.0			
Neutral	216 (33.5)	147 (29.4)	69 (47.6)	2.64 (1.60, 4.34)	< 0.001*	1.01 (0.49, 2.08)	0.982
Agree	335 (51.9)	311 (62.2)	24 (16.6)	16.04 (8.97, 28.69)	< 0.001*	1.34 (0.57, 3.13)	0.501

### 3.4 Multivariable regression model

An overall logistic regression model using sex, ethnicity, race, education level, household income, and beliefs regarding the safety, protectiveness, transparency, and timeliness of the authorization process of the COVID-19 vaccines to predict current plans to obtain the COVID-19 vaccine was created. The model was statistically significant [χ^2^(19) = 314.41, *p* < 0.001], and findings are summarized in Table 2. There were no associations between a respondent’s ethnicity, level of education obtained, income level, or perceptions regarding the timeliness of the authorization process of the COVID-19 vaccines and their plans to receive the COVID-19 vaccine. After adjusting for other variables, findings indicated that males had a 104.6% increased odds of planning to obtain the COVID-19 vaccine as compared to females (POR: 2.05, *p* = 0.014, 95% CI: 1.16, 3.62).

Results suggest that the odds of planning to obtain the COVID-19 vaccine among respondents who agreed with the statement, “the research and development process has produced COVID-19 vaccines that are safe,” were 8.71 times the odds compared with respondents who disagreed with the statement (POR: 8.71, *p* = 0.002, 95% CI: 2.25, 33.66) after adjusting for other variables. Respondents who agreed with the statement, “the research and development process has produced COVID-19 vaccines that will protect me,” had 13.45 times the odds of planning to obtain the COVID-19 vaccine than respondents who did not agree (POR: 23.45, *p* = 0.001, 95% CI: 3.10, 58.34) while controlling for other variables in the model. Respondents who agreed with the statement, “the FDA emergency use authorization process for approving COVID-19 vaccines is transparent,” had 1.33 times the odds of planning to obtain the COVID-19 vaccine than respondents who did not agree (POR: 1.33, *p* = 0.024, 95% CI: 1.15, 7.15) after adjusting for other variables. Plans to obtain the COVID-19 vaccine did not differ between respondents who disagreed with the statement “the time it took to authorize COVID-19 vaccines was appropriate,” and those who agreed (*p* = 0.50) or were neutral (*p* = 0.98). Results of the adjusted analyses are shown in Table 2.

## 4 Discussion

This study analyzed factors that influence an individual’s plan to be vaccinated against COVID-19. We used selected demographic variables and perceptions regarding the safety, protectiveness, and authorization process of the mRNA COVID-19 vaccines as predictors for plans to receive the vaccine using logistic regression modeling.

Interestingly, while several demographic variables were independent predictors of the outcome in the bivariate analysis, only sex at birth, race, and perceptions regarding the safety, protectiveness, and authorization process of the COVID-19 vaccines remained significant predictors of plans to receive the vaccine in the adjusted model. Other studies exploring the relationship between COVID-19 vaccine perceptions and vaccination intent found similar results, indicating that vaccine safety, development time, and efficacy are associated with an individual’s willingness to be vaccinated (13, 14). Importantly, while the majority of respondents within these studies reported intentions to be vaccinated, concerns about the vaccines remained. In a prior qualitative study, we report on underlying reasons influencing vaccination intentions, which illustrate some respondents’ reservations regarding vaccine safety, clinical trials, and the lack of data on the effectiveness of COVID-19 vaccines (15). Qualitative and quantitative studies provide evidence, documentation, and insights into factors that influence vaccination intent and key barriers affecting vaccine hesitancy during a public health crisis.

Our results expand on a body of literature that has analyzed the influence of sociodemographic characteristics on COVID-19 vaccination (12, 16–19). Our findings are an important contribution to the literature because this study goes beyond the use of sociodemographic factors to identify characteristics related to vaccine hesitancy. Rather, this study assesses how external non-sociodemographic factors influence vaccination intent and vaccine hesitancy among a discrete population of individuals who have health insurance in the United States. Additionally, data were collected in the first week of COVID-19 vaccine availability for the general public in Central Texas. We believe we are the only research group in Texas to have collected such data at this critical time of vaccine availability. Data obtained during ongoing public health emergencies can be used to identify and mitigate real-time concerns regarding novel vaccines to support their uptake. Therefore, when possible, public health institutions should consider obtaining real-time data regarding perceptions of vaccine development, safety, and trust to inform and modify public health strategies used to address vaccine hesitancy throughout the pandemic period. The use of real-time data could prove critical in reaching vaccine-induced herd immunity thresholds. Data collected during post-pandemic studies regarding vaccine concerns can be affected by selection and information biases.

The COVID-19 pandemic prompted the introduction of mRNA technology in the first human vaccines approved through an expedited EUA process. Public perception regarding this new vaccine, its technology, development, approval process, and adoption has set a precedent that public health officials can consider for future vaccination efforts involving mRNA and other novel technologies. In Texas, the majority of vaccine uptake occurred during the first 6 months of vaccine availability, when state-level COVID-19 restrictions and recommendations were in full effect. It is estimated that full-dose vaccination reached 57% by the end of the first year of vaccine availability, with an additional 7% occurring in the subsequent 17 months prior to May 2023, when the Texas Department of State Health Services ended weekly data releases (20–22). This pattern of vaccine uptake illustrates the importance of achieving vaccine acceptance at an early stage, as it presents an opportunity to reach vaccine-induced immunity goals while the media and public interests are focused on mitigating the pandemic, and vaccination campaigns are most likely to be impactful.

Looking ahead, public health officials should consider the precedent set by vaccination efforts involving new vaccine technology similar to mRNA, with the understanding that public perception may not favor such vaccines and that vaccination expectations (in support of reaching the vaccine-induced immunity threshold) may need to be realigned to reflect the real-world vaccination rates that will occur. If the COVID-19 pandemic serves as any indication, community adoption of future vaccines incorporating new technology will need to overcome barriers posed by perceptions of safety, protectiveness, and trust in the vaccine development process.

The COVID-19 pandemic provided many lessons. One lesson is the importance of aligning public opinion with messaging from public health organizations and political leaders to build trust in vaccines. Robust strategies for disseminating important information regarding the research, development, and approval process of vaccines, as well as news on technological vaccine advances like mRNA technology, are vital to acquiring community-level support for vaccines and improving vaccine uptake during public health emergencies. Public health leaders and stakeholders should focus on building general vaccine acceptance, as developing a communal sense of ingrained trust in vaccines is best for the long-term adoption of healthy practices—particularly as new vaccines and technologies are developed.

A second lesson regards non-governmental organizations and their role in public health, which merits a call to action in preparation for future public health emergencies. Health insurance providers, like Sendero, have access to defined populations that can provide valuable data and insight to aid public health officials in understanding barriers to public health response strategies, including vaccination campaigns. This capability allows non-governmental organizations to support and participate in public health efforts in their communities. We encourage other organizations and systems with similar capabilities, data resources, and positions within their communities to partake in activities that can assist public health preparedness, response, and the development of a more connected public health system.

### 4.1 Limitations

This study has several limitations. First, respondents to this survey had already shown a willingness to participate in a survey on COVID-19-related questions and indicated a willingness to participate in a follow-up survey. As such, these individuals may differ from non-responders in ways related to health-seeking behavior. Second, the findings of this study may not be generalizable beyond the specific population studied—individuals insured by an ACA health plan—who may differ in demographic or socioeconomic characteristics from uninsured individuals or those insured through public programs, commercial insurers, or other schemes. Third, our study did not measure historical vaccine hesitancy among the study population; therefore, it is possible that some individuals exhibited a general hesitancy toward vaccines rather than hesitancy specific to the COVID-19 vaccines. Finally, because this survey was administered by a health insurance provider to its members, respondents may have been more inclined to respond positively to statements regarding the vaccine or plans to obtain the vaccine.

## 5 Conclusion

Future pandemics involving a vaccine-preventable disease will require a public health emergency response similar to that of the COVID-19 response. The rapid development and deployment of safe and effective vaccines using both new technologies and the emergency use authorization process may be necessary to disrupt the spread of disease, reduce the burden of disease on the healthcare system, and save lives. Reducing barriers to the uptake of novel EUA-approved vaccines and improving the likelihood of vaccine uptake requires public trust in the agencies with the authority and responsibility to develop, approve, and distribute such vaccines. Assessing perceived safety, perceived effectiveness, the transparency of the EUA process, and timeliness of vaccine development and distribution provides valuable information to public health professionals working to address barriers related to vaccine hesitancy during a pandemic. Assessment, followed by targeted public health messaging and cues-to-action that address specific barriers associated with research and development of novel vaccines, may help reduce vaccine hesitancy, improve trust in vaccine technologies, and promote vaccine uptake during future disease outbreaks.

## Data Availability

The raw data supporting the conclusions of this article will be made available by the authors, without undue reservation.
